# Modified Maximum Entropy Method and Estimating the AIF via DCE-MRI Data Analysis

**DOI:** 10.3390/e24020155

**Published:** 2022-01-20

**Authors:** Zahra Amini Farsani, Volker J. Schmid

**Affiliations:** 1 Statistics Department, School of Science, Lorestan University, Khorramabad 68151-44316, Iran; 2 Bayesian Imaging and Spatial Statistics Group, Institute of Statistics, Ludwig-Maximilians-Universität München, Ludwigstraße 33, 80539 Munich, Germany; volker.schmid@lmu.de

**Keywords:** kinetic model, modified maximum entropy method, arterial input function, optimization method

## Abstract

**Background**: For the kinetic models used in contrast-based medical imaging, the assignment of the arterial input function named AIF is essential for the estimation of the physiological parameters of the tissue via solving an optimization problem. **Objective**: In the current study, we estimate the AIF relayed on the modified maximum entropy method. The effectiveness of several numerical methods to determine kinetic parameters and the AIF is evaluated—in situations where enough information about the AIF is not available. The purpose of this study is to identify an appropriate method for estimating this function. **Materials and Methods**: The modified algorithm is a mixture of the maximum entropy approach with an optimization method, named the teaching-learning method. In here, we applied this algorithm in a Bayesian framework to estimate the kinetic parameters when specifying the unique form of the AIF by the maximum entropy method. We assessed the proficiency of the proposed method for assigning the kinetic parameters in the dynamic contrast-enhanced magnetic resonance imaging (DCE-MRI), when determining AIF with some other parameter-estimation methods and a standard fixed AIF method. A previously analyzed dataset consisting of contrast agent concentrations in tissue and plasma was used. **Results and Conclusions**: We compared the accuracy of the results for the estimated parameters obtained from the MMEM with those of the empirical method, maximum likelihood method, moment matching (“method of moments”), the least-square method, the modified maximum likelihood approach, and our previous work. Since the current algorithm does not have the problem of starting point in the parameter estimation phase, it could find the best and nearest model to the empirical model of data, and therefore, the results indicated the Weibull distribution as an appropriate and robust AIF and also illustrated the power and effectiveness of the proposed method to estimate the kinetic parameters.

## 1. Introduction

Determining the probability density function of a random variable based on observations is a major and old issue in statistics. In recent years, various parametric and non-parametric methods have been introduced for the estimation of the probability density function for a random variable based on observations, but there is very limited work reported on the optimization methods. The maximum entropy method (MEM) is one of the major methods for estimating and determining the probability density with a high level of accuracy and efficiency and minimum bias. It is applied to gain the unknown density via resolution of an optimization problem. The principle of maximum entropy, as a method of statistical inference, is due to Jaynes [1]. His idea is that this principle leads to the selection of a probability density function that is consistent with our knowledge and introduces no unwarranted information. Any probability density function satisfying the constraints that has smaller entropy will contain more information (less uncertainty), and thus says something stronger than what we are assuming [1,2,3,4]. Entropy maximization or related concepts has been frequently utilized in the past ten years to analyze large biological datasets in various fields. These fields range from determining macromolecular interactions and structures [5,6,7,8,9,10,11,12,13,14,15,16,17,18,19,20] to inferring signaling [21,22,23,24,25] and regulatory networks [26,27,28] and the coding organization in neural populations [28,29,30,31,32,33,34,35,36,37,38,39,40,41,42,43] based on DNA sequence analyzes (the detection of specific binding sites, for instance) [42,43,44,45,46]. MEM is a powerful vehicle to reconstruct images based on various datasets. It is also commonly used in radio astronomical interferometry, which deals routinely with images with high dynamic range and up to a million pixels [47,48].

In this paper, a concise and basic introduction to entropy maximization and its applicability for deriving models from biological datasets—especially in kinetic models and image processing via DCE-MRI—is provided. DCE-MRI is a fast and noninvasive method for quantitative perfusion analysis in soft tissue, using a contrast agent (CA). It is used extensively for the analysis of microvascular blood in a variety of clinical usages like detecting, characterizing, and therapeutic monitoring of different diseases [49,50,51,52,53,54]. In DCE-MRI, quantitative analysis is often applied on a whole tumor region of interest (ROI) [55], in which the contrast agent concentration time curve for all voxels in the tumor is used to estimate a single set of kinetic parameters (like—volume transfer constant between blood plasma and extracellular extravascular space per minute—and—rate constant between extracellular extravascular space and blood plasma per minute [56]) for each patient study.

In recent years, several approaches have been proposed to quantify the perfusion of CA into tissues and to estimate the related parameters of perfusion (indices) from concentration-time or signal-curves [57,58,59,60]. In DCE-MRI, quantification of the perfusion involves measuring the concentration of the CA in tissue over time. This time curve is then modeled using kinetic processes, where the kinetic parameters are of clinical interest [55]. The kinetic model of the tissue is explicit as an ordinary-differential equation, dissolved analytically result in a nonlinear format in the contrast agent concentration [61,62,63,64,65,66,67,68,69].

One fundamental necessity for the Tofts pharmacokinetic analysis is the knowledge of the arterial input function (AIF), that is the time curve of CA concentration on the left-side ventricular blood pool. Although the AIF itself is not clinically relevant, its correct determination is very important for the correct estimation of kinetic parameters [62,70]. Since the obtained rate constants are heavily dependent on the AIF [71,72,73,74,75], an accurate and precise measurement is necessary for their absolute and reliable quantification. Instead, a simplified method, such as a population averaged AIF can be used. However, (large) variabilities in cardiac output—between patients and within patients over time—are no longer taken into account with this method. If this variability in cardiac output can be accounted for by precise measurement of the AIF, the accuracy and repeatability of the kinetic parameters should be superior over use of a population averaged AIF. Some researchers have shown that a population averaged AIF can result in better repeatability [76,77], whereas others report the opposite [78,79]. It is possible that repeatability depends on the imaged body part and imaging sequence parameters, but also on the choice of the artery for AIF measurement.

However, in many imaging applications, e.g., for patients with breast cancer, it is not possible to directly measure the AIF from imaging, as no large vessel is in the field of view. Thus, assumed AIFs from the literature are often used, e.g., bi-exponential functions with parameters derived by [80] or [81] or a mix of the two Gaussian with an exponential [61,62,63,64,65,66,67,68,69,76].

We have previously developed a method for estimating the AIF and the kinetic parameters in DCE-MRI [82]. This method was developed in response to a need in the medical imaging community for the objective comparison of estimations made using different statistical methods, for example, the Bayesian method and MLE. The main problem of our previous algorithm was the dependence of Newton’s method on the starting point, which was a uniform random number.

The previous algorithm was the maximum entropy method in combination with Newton’s method in order to estimate the AIF using the CA time curve data in plasma, also called “blind estimation” of the AIF and the maximum a posterior approach (MAP) to determine the kinetic parameters [82]. In this paper, we propose an improved algorithm for blind AIF estimation using a mixture of the maximum entropy method (MEM) and teaching-learning based optimization in the step of λ’s estimation for assessing observer performance in the classification tasks using available information. This circumvents issues with the random start points in the previous algorithm. This approach works on the effect of the influence of a teacher on students. Like other nature-inspired algorithms, TLBO is also a population-based method and uses a population of solutions to proceed to the global solution. The population is considered as a group of learners or a class of learners. The process of TLBO has two parts: the first part consists of the ‘Teacher Phase’ and the second part consists of the ‘Learner Phase’. ‘Teacher Phase’ means learning from the teacher, and ‘Learner Phase’ means learning by the interaction between learners [83,84]. The proposed algorithm is, therefore, more robust. It finally proposed the Weibull distribution in between all other selective models as a model for the AIF via MEM approach. We performed extensive studies using empirical data to better understand the performance of our method. In addition, a comparison was conducted among four other different estimation methods in DCE-MRI dataset, and the new recommended method results were compared with the previous work [55,82].

The rest of this paper is structured as follows: Section 2 describes the basic structure of the proposed method for DCE-MRI analysis: the proposed modified maximum entropy method (MMEM), TLBO algorithm, and MAP. Section 3 includes alternative approaches for estimating of the parameters. Section 4 gives an example to show the step-by-step analysis of the dataset. Section 5 contains the application of a complete DCE-MRI study using the proposed method and their evaluations. Section 6 concludes the aim of the present work.

### Data Description

As an example data set, we use a previously analyzed breast cancer data set [55]. The data were provided by the Paul Strickland Scanner Center at Mount Vernon Hospital in Northwood, UK. Pre-treatment DCE-MRI scans of twelve patients were available. In each case, 46 images were recorded every 11.9 s after administration of the contrast agent Gadolinium-DTPA. For the calculation of $T_{1}$ values, we used a two-point measurement with calibration curves as described in [85,86]. In DCE-MRI, $T_{1}$ is the relaxation time, also known as the spin-lattice relaxation time. In addition, it is a measure of how quickly the net magnetization vector recovers and its ground state or it is the time constant for regrowth of longitudinal magnetization. The $T_{1}$ values are computed as a ratio of a $T_{1}$-weighted fast low-angle shot (FLASH) image and a proton-density-weighted FLASH image.

To measure contrast agent concentration $C_{t}\left(t\right)$, the signal intensity is converted to $T_{1}$ relaxation time values using $T_{1}$-weighted images, proton density weighted images and data from calibration phantoms with known $T_{1}$ relaxation times [87]. The Gd-DTPA concentration can then be computed via $C_{t}\left(t\right)=\frac{1}{r_{1}}\left[\frac{1}{T_{1}\left(t\right)}-\frac{1}{T_{10}}\right],$ where $T_{10}$ is the $T_{1}$ value without contrast, computed as mean value of the first four images, and $r_{1}=4.24\;l/s/\mathrm{mmol}$ is the longitudinal relativity of protons in vivo due to Gd-DTPA. The imaging parameters of the T1-weighted FLASH images were TR = 11 ms, TE = 4.7 ms, α = 35, the parameters of the proton density-weighted image were TR = 350 ms, TE = 4.7 ms, α = 6. Field of view was the same for all scans, 260×260×8 mm per slice, so voxel dimensions are 1.016×1.0168 mm. A scan includes three sequential slices of 256×256 voxels and one slice placed in the contralateral breast as control, which we do not use for our analysis. A dose of D = 0.1 mmol per kg body weight of Gd-DTPA was injected after the fourth scan via a power injector with 4 mL/s with a 20 mL saline flush also at 4 mL/s.

**Figure 1:**
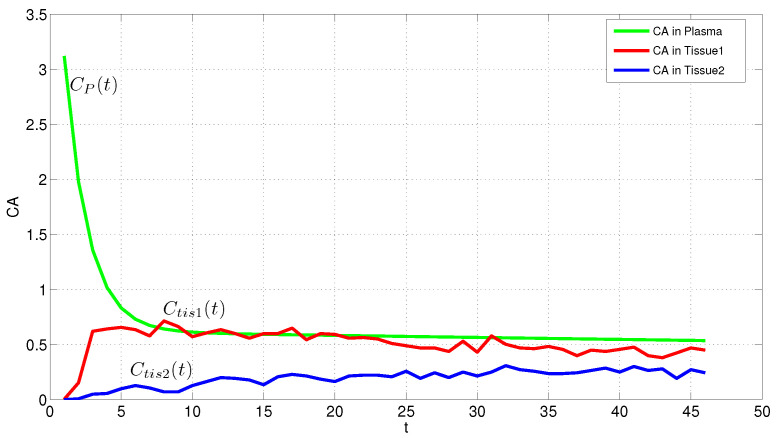
Empirical PDF of the contrast agent in plasma ($C_{p}\left(t\right)$) named empirical AIF and in tissue ($C_{tis}\left(t\right)$) for two patients.

Figure 1 shows the empirical model of data for two patients ${\vec{C}}_{tis-1}$, ${\vec{C}}_{tis-2}$ and ${\vec{C}}_{p}$ based on the time *t* using Kernel distribution which actually finds an empirical density function of the sample data (See: Using “KernelDistribution” Objects and “ksdensity” in Matlab). In statistics, kernel density estimation is a non-parametric way to estimate the probability density function of a random variable and it is a smoothing function that determines the shape of the curve used to generate the pdf, and a bandwidth value that controls the smoothness of the resulting density curve [88]. This is the primary model of data $C_{p}\left(t\right)$ changing the inverse problem to forward. It is not clear the sample data belongs to which family of distributions, it is the main issue in here we try solving here by the maximum entropy method.

## 2. Theory and Methods

In this study, we estimated different probability density functions for AIF using our proposed algorithm. The accurate estimated model of AIF is the cornerstone of the present work, because its correct determination is very important for the correct estimation of kinetic parameters. In the previous study [82], we have estimated the gamma and exponential distributions using the maximum entropy method and Newton’s approach. Both models were acceptable approximated AIF in comparison to the literature models.

Here, the modified algorithm examines different moments constraints in MEM to build the best probability model fit to data. In addition, besides TLBO, the various parameter estimation methods then suggest the estimated parameters which help to find more appropriate AIF. Kinetic parameters are estimated in the next step via MAP.

### 2.1. Kinetic Model

The kinetic process in the tissue can be modeled using an ordinary-differential equation, dissolved analytically resulting in a nonlinear model for the contrast agent concentration [61,62,63,64,65,66,67,68,69]. In this study, we adopted the commonly used pharmacokinetic model [89] that assumes the CA resides in and exchanges between two compartments in the tissue: the vascular space and EES.

Considering the kinetic properties of the contrast agent (CA) in the tissue of interest ($C_{tis}$) using DCE-MRI, we apply the differential equation system as follows:

(1) $$\frac{dC_{tis}\left(t\right)}{dt}=K_{1}C_{p}\left(t\right)-K_{2}C_{tis}\left(t\right),$$

in which $C_{p}\left(t\right)$ is the CA concentrations in the vascular blood pool, that is, the arterial input function AIF. Both $K_{1}$ and $K_{2}$ are the rate constants of the CA exchanges between extravascular-extracellular space (EES) and plasma. Subject to $C_{p}\left(0\right)=0$, Equation (1) can be solved with the following result

(2) $$C_{tis}\left(t\right)=K_{1}\int_{0}^{t}C_{p}\left(u\right)e^{-K_{2}\left(t-u\right)}du.$$

This equation has been used to analyze MR data in a number of studies [81]. Murase [90] proposed a different way to solve Equation (1) using discretization:

(3) $$C_{tis}\left(t\right)=K_{1}\int_{0}^{t}C_{p}\left(u\right)du-K_{2}\int_{0}^{t}C_{tis}\left(u\right)du,$$

This can be written in matrix form as follows:

(4) $$\vec{C}=\vec{A}\times \vec{K},$$

where matrix $A_{n\times 2}$: $\vec{A}={\left\{A\left(1\right),\ldots ,A\left(n\right)\right\}}^{\prime }$ for each row of I=1,2,⋯,n:

(5) $$\begin{array}{c} A\left(I\right)=(\int_{0}^{t_{I}}C_{p}\left(u\right)du,\;\;-\int_{0}^{t_{I}}C_{tis}\left(u\right)du), \end{array}$$

(6) $$\vec{K}=\left(\begin{array}{c} K_{1}\\ K_{2} \end{array}\right)$$

and

(7) $$\vec{C}=\left(\begin{array}{c} C_{tis}\left(t_{1}\right)\\ C_{tis}\left(t_{2}\right)\\ \vdots \\ C_{tis}\left(t_{n}\right) \end{array}\right).$$

From the mathematical view, when $C_{tis}\left(t_{i}\right)$ and $C_{p}\left(t_{i}\right)$ are measured, it is possible to use conventional linear least-squares (LLSQ) method to determine $\vec{K}$ and the trapezoidal rule for the elements of $\vec{A}$. Unfortunately, this method measures approximate values for the kinetic parameters. A great number of image processing problems can be presented as inverse problems. In here the linear system of equations which are obtained after the discretization of the integral equations which arises in various tomographic image restoration and reconstruction concerns are considered: Therefore, we write Equation (2) as follows:

(8) $$y_{tis}\left(t_{i}\right)=A\left(i\right)\vec{K}+\varepsilon_{i}\;\;\;\;\;\;\varepsilon_{i}∼N\left(0,\sigma^{2}\right)$$

where $y_{tis}\left(t_{i}\right)$ is the observed tissue concentration at time $t_{i}$ and the measurement uncertainty (noise) which is assumed to be additive, centered, white, Gaussian and independent of *K* [91].

For that, the estimation procedure of the analysis here includes Bayesian methods, which its advantage is to overcome the integration process involved in estimating the model parameters. Therefore, Bayesian methods can provide exact estimates of the model parameters, not approximating them. In Bayesian statistics, parameters are viewed as random variables. Each parameter involved in a Bayesian model has a distribution attached to it in order to express the uncertainty about its true value. The distribution is named the prior distribution. They represent the prior knowledge about the parameter of interest, which is often obtained from historical data (data-based priors) [92,93].

### 2.2. Maximum a Posterior Approach

Image reconstruction belongs to the class of ill-posed inverse problems of mathematical physics [94]. In 1967, the physicist V. Turchin suggested using the Bayesian method of maximum a posteriori (MAP) for solving inverse ill-posed problems with stochastic data, naming this approach ‘statistical regularization’ [95]. Bayesian maximum a posteriori (MAP) approaches can be used to solve ill-posed problems as they arise in image reconstruction [96,97,97]. The solution of MAP obviously depends on the priori models. The main challenge of the Bayesian method is how to determine the a priori probability distribution of the studied image and specify its parameters using its data. In here, we assume a form of a priori information named entropy-based prior, which relies on the principle of entropy. Such approaches have been successfully used in the fields of plasma-tomography, X-ray, radio, and gamma-astronomy [93,96,97,98]. To estimate the kinetic parameters, we consider the general form of Equation (8) in the following as proposed by [92,93]. Estimating the positive-vector *x* (the pixel-intensities in an object) subject to a vector of measurement *y* (e.g., a degraded-image or the projections of an object) and a linear-transformation *A* which relates both vectors by

(9) y=Ax+b,

where *b* is uncorrelated noise with normal distribution, and zero-mean. We consider only approximate information about the variance of the noise $\Sigma^{2}$ and general information about the object.

To estimate the unknown vector $\vec{x}$, we use a Bayesian approach. Given the probability density functions (pdf) f(x) and f(y|x) and f(y) we obtain the pdf of the conditional distribution of *x* subject to *y* using the Bayesian formula [99]:

(10) $$f\left(x|y\right)=\frac{f(y|x)\cdot f(x)}{f(y)}.$$

The MAP estimator $\hat{x}$ maximizes the posterior pdf f(x|y) obtained by Bayes formula. In the Equation (10), f(y) is independent of *x*, f(y|x) relates to the noise probability distribution and finally, f(x) is a prior distribution on *x*.

If we are not able to directly determine f(x) and f(y|x), we can apply the maximum entropy method. For the MEM, knowledge of some constraints on f(x) can be used. Among all probability distributions satisfying these constraints, we select the one which has maximum entropy [92,93,93], see Section 2.3. To determine f(y|x), the noise pdf, we have

$$\begin{array}{ccc} f(y|x)\; & \approx & \exp[-T(x)],\\ T(x) & = & {\left[y-Ax\right]}^{t}\left[y-Ax\right]/\sigma^{2}. \end{array}$$

A possible way to select a priori-distribution, f(x), is to apply the MEM where the general model is in the form of the exponential family. The advantage of applying MEM for finding a priori is, that this method is the most objective, and maximally uncommitted [99].

### 2.3. Maximum Entropy Method

The maximum entropy principle allows one to determine the least biased probability distribution function when the information available is limited by some macroscopic constraints [100]. The MEM determines the randomness of the primary data by the concept of information entropy. It is the mathematical expectation of the uncertainty associated with an outcome in terms of its occurrence probability. It is suggested that the most likely probability distribution should be the one that maximizes the information entropy. Maximizing Shannon’s entropy is the basic idea of MEM [4]:

(11) h(X)=−∫f(x)logf(x)dx,

subject to known constraint, typically moment constraints

(12) $$E\left(\phi_{k}\left(x\right)\right)=\int \phi_{k}\left(x\right)f\left(x\right)dx=\mu_{k}.$$

Here $\phi_{0}\left(x\right)=1$, and $\phi_{k}\left(x\right)$, k=0,…,N are N+1 known-functions. These could be, for example, $x^{n}$,log(x), xlog(x) or trigonometric or geometric functions. The idea is to assign the appropriate known-function relating the ME distribution to the exponential family via the mentioned constraints [2,3,101]. Using the method of Lagrange multipliers method, where the objective function is Shannon’s entropy Equation (11), J(f) is as follows:

(13) $$J\left(f\right)=-\int f\left(x\right)logf\left(x\right)dx+\lambda_{0}\int f\left(x\right)dx+\sum_{k=1}^{N}\lambda_{k}\int f\left(x\right)\phi_{k}\left(x\right)dx.\;$$

For obtaining f(x), we differentiate *J* subject to f(x):

(14) $$\frac{\Delta J(f)}{\Delta f(x)}=-logf\left(x\right)-1+\lambda_{0}+\sum_{k=1}^{N}\lambda_{k}\phi_{k}\left(x\right)=0.\;$$

Adopting Taylor’s theorem (based on using a Taylor series approximation), the required expected values $\mu_{1},\ldots ,\mu_{m}$ can be obtained numerically from the data set [101]. Applying an optimization method with Shannon’s entropy as the objective function, and setting Equation (14) equal to zero, the general form of ME distribution will be [4]:

(15) $$f\left(x\right)=e^{-\sum_{k=0}^{N}\lambda_{k}\phi_{k}\left(x\right)},\;\;x\in S,\;$$

where $\lambda_{k}$ can be selected such that, f(x) in Equation (15) satisfies the known constraints in Equation (12). The parameters $\lambda =[\lambda_{0},\cdots ,\lambda_{N}]$ are determined to fit an appropriate class of the ME distributions. To determine the N+1 unknown-parameters, the set of N+1 nonlinear equations are solved as follows (1≤k≤m):

(16) $$G_{k}\left(\lambda \right)=\int \phi_{k}\left(x\right)e^{-\sum_{k}\lambda_{k}\phi_{k}\left(x\right)}dx=\mu_{k}.$$

### 2.4. Teaching-Learning Based Optimization

To solve Equation (13) we suppose a population base algorithm named teaching-learning based optimization (TLBO), which resolves the problem of random starting points. Instead, it can measure the mean of all possible parameter estimations in order to fit a better model to data. The idea of this algorithm is to assume the relationships between a teacher and all learners in a classroom [83]. It considers the same probability for learners to get information from others. TLBO has two steps to find the best solutions, which named teaching and, learning. The most important features of this algorithm is the easy implementation, and rapid convergence [102].

It considered the population as a group of students and their related subjects are the design variables of the problem. In TLBO, the different presented courses for students are supposed to be different variables and their scores are like the objective function. It is very important for teachers to share their knowledge between students to improve the class’s level of knowledge, then it can cause achieving acceptable scores by students based on their talents. The teacher is assumed to be the most informed person in the classroom, which distributes her/his knowledge to the students. For that, the teacher will be the best solution (the best person in the population) among all. There is a fact that the student’s level of knowledge highly depends on the teacher’s quality of the teaching, and the quality of the others in the class. Therefore, the two main steps of learning for the students are implemented After the creation of the initial population and calculation of the objective value for each individual as follows:

#### 2.4.1. Teacher Phase

In this step, the teacher tries to improve the mean scores of the students subject to her/his situation. The random procedure here is to produce a new solution instead of the old one:

(17) $$X_{new,D}=X_{old,D}+r\left(X_{teacher,D}-T_{F}M_{D}\right)$$

where, *D* shows the number of courses, $X_{old,D}$ (a vector 1×D) is the old solution, when there is no contribution between the students to improve their knowledge, and it includes the results of each specific course, a random number *r* is in [0,1], $X_{teacher,D}$ is the best solution of the whole population, $T_{f}$ is a teaching factor changes from 1 to 2 randomly with the same probability, and $M_{D}$ is a vector ( 1×D) involving the mean values of the classroom results for each specific course. The new solution $X_{new,D}$ is accepted if it is better than the old one [102].

#### 2.4.2. Learner Phase

To improve the knowledge of each student when randomly cooperating with other students, Equation (18) is implemented to all of them, so that, one can achieve the new information in the situation when the other learner has more knowledge than her/him.

(18) $$X_{new,i}=X_{old,i}+r_{i}\left(X_{j}-X_{k}\right)$$

where i=1,2 is the total number of solutions, $X_{old,i}$ when there is no cooperation with other students, $r_{i}$ is a random number in the range [0,1], and $X_{j}$ and $X_{k}$ are two learners randomly chosen with j≠k and in which $X_{j}$ presents a better objective value than $X_{k}$. The solution $X_{new,i}$ is accepted if it is better than the old solution $X_{old,i}$.

### 2.5. Implementation

We have implemented the code for the proposed method in MATLAB. These are the main steps of the algorithm:

(1) Determining $\phi_{k}\left(x\right)$ and their numerical expectations using dataset via Taylor’s theorem [4],
(2) Using TLBO (or an alternative optimization method, see below) to determine the unknown function with the Shannon’s entropy as target function. The general form is given in Equation (15), ($f_{C_{tis}}\left(t\right)$ and $f_{C_{p}}\left(t\right)$),
(3) Applying the proposed method to find $\lambda_{k}$ in which f(x) (Equation (15)) matches the constraints in (Equation (12)), (${\hat{f}}_{C_{tis}\left(t\right)}$ and ${\hat{f}}_{C_{p}}\left(t\right)$),
(4) Estimating the kinetic parameters $\vec{K}$, we replace ${\hat{f}}_{C_{tis}}\left(t\right)$ and ${\hat{f}}_{C_{p}}\left(t\right)$ in Equation (8) and resolve them via MAP,
(5) Using the Kullback–Leibler divergence $D_{K-L}\left(f||g\right)$ to check the accuracy of the estimated AIF, ${\hat{f}}_{C_{p}}\left(t\right)$ in comparison with the empirical distribution of dataset $g(C_{p})$,
  (19) $$D_{K-L}\left(\overset{}{\hat{f}}||g\right)=\int_{s}{\hat{f}}_{C_{p}}\left(t\right)log\frac{{\hat{f}}_{C_{p}}\left(t\right)}{g(C_{p})}dt.$$
(6) With the predicted values ${\hat{x}}_{1},\cdots ,{\hat{x}}_{m}$ and the observed values $x_{1},\cdots ,x_{m}$:
  (20) $$RMSE={\left[\frac{1}{m}\sum_{i=1}^{m}{\left(x_{i}-{\hat{x}}_{i}\right)}^{2}\right]}^{1/2},$$
  (21) $$\chi^{2}=\frac{\sum_{i=1}^{m}{\left(x_{i}-{\hat{x}}_{i}\right)}^{2}}{m-n},$$
  (22) $$R^{2}=1-\frac{\sum_{i=1}^{N}{\left(x_{i}-{\hat{x}}_{i}\right)}^{2}}{\sum_{i=1}^{N}{\left(x_{i}-\bar{x}\right)}^{2}},$$

## 3. Alternative Parameter Estimation

### 3.1. Weibull Distribution

The Weibull distribution is widely used in reliability and life data analysis due to its versatility. It is also established a close approximation to the probability laws of various natural-phenomena. The pdf of the Weibull distribution has two parameters:

(23) $$f\left(x\right)=\frac{k}{c^{k}}x^{k-1}e^{-{\left(\frac{x}{c}\right)}^{k}},$$

where *k* is the shape and *c* is the scale parameter [103,104].

Using the Weibull model, we can also use different approaches to determine the parameters *k* and *c*:

### 3.2. Methods of Moments

The method of moments (MM) calculates the first and second moments to estimate shape and scale parameters. The algorithm relies on the mean, variance of the gamma-function of (1+1/k) [105]. The sample mean and standard error are

(24) $$\bar{x}=c\Gamma \left(1+1/k\right),{\sigma =c(\Gamma \left(1+1/k\right)-\Gamma^{2}\left(1+1/k\right))}^{1/2},$$

where $\Gamma \left(x\right)=\int_{0}^{\infty }t^{x-1}e^{-t}dt$ is the gamma function.

### 3.3. Empirical Measurement Method

The empirical measurement method is a special case of MM [105,106,107]:

(25) $$k={\left(\frac{\sigma }{\bar{x}}\right)}^{-1.086},c=\frac{\bar{x}}{\Gamma (1+\frac{1}{k})},$$

where σ is the sample standard deviation.

### 3.4. Maximum Likelihood Method

For the maximum likelihood estimator (MLE), an iterative algorithm can be used. With *n* the number of non-zero data points, the shape and scale parameters (k,c) are by iteratively solving

$$k={\left(\frac{\sum_{i=1}^{n}x_{i}^{k}\ln\left(x_{i}\right)}{\sum_{i=1}^{n}x_{i}^{k}}-\frac{\sum_{i=1}^{n}\ln\left(x_{i}\right)}{n}\right)}^{-1},$$

and

$$c={\left(\frac{1}{n}\sum_{i=1}^{n}x_{i}^{k}\right)}^{1/k},$$

### 3.5. Modified Maximum Likelihood Method

When the data is available in the form of the frequency distribution, we can apply the modified maximum likelihood method (MMLE).

(26) $$k={\left(\frac{\sum_{i=1}^{n}x_{i}{}^{k}\ln\left(x_{i}\right)P\left(x_{i}\right)}{\sum_{i=1}^{n}x_{i}{}^{k}P\left(x_{i}\right)}-\frac{\sum_{i=1}^{n}\ln\left(x_{i}\right)P\left(x_{i}\right)}{P(x\geq 0)}\right)}^{-1},\;\;\;c={\left(\frac{1}{P(x\geq 0)}\sum_{i=1}^{n}x_{i}{}^{k}P\left(x_{i}\right)\right)}^{1/k},$$

where $P(x_{i})$ represents the frequency of data $x_{i}$, *n* the number non-zero data points, and P(x≥0) the probability of the random variable equal or exceeding zero. In Equation (26), *k* can be resolved iteratively, then *c* can be solved explicitly [108,109].

### 3.6. Non-Linear Least Squares Method

For the non-linear least squares method (NLSM) the observations are ordered in an ascendant form, and coupled to the failure probabilities, gained by the estimators. The Gauss–Newton’s algorithm is used to gain the best-fitted curve of a Weibull model [110].

Based on the individual results by the method of maximum likelihood, modified maximum likelihood, and least-squares regression, Seguro and Lambert [111] concluded that maximum likelihood or modified maximum likelihood proposed here provided more reasonable and accurate values for parameter estimation of Weibull distribution than least-squares regression. Later it is proven once again by Cook [112].

## 4. Example of Application

In this section, we show the application of the modified maximum entropy method (MMEM). Due to the limitation of length, this paper only provides a brief description of formulas and fitting curves.

An additional challenge is the fact that usually there are an infinite number of statistical models that are consistent with a given set of global properties measured from data. Therefore, one needs an additional criterion to decide which model to use. The benefit of using the maximum entropy method is to find the simplest model with the lowest bias, which maximizes the entropy. For the same dataset, there could be many complicated models to describe data, but finding the best fitted model with a few numbers of constraints, a few steps of computations, and finally, with the known family of distribution, is the advantage of choosing this method. For that, the maximum entropy method, searches, examines and, applies different moment constraints (see Section 2.3) [3], and adopts the minimum number of them to form an appropriate probability density model for the sample data. There could be a large difference between the maximum entropy estimated model with two constraints and three constraints, but for the estimated ME model with four or five cases, it would not be a big difference in the estimated models. In other words, there is no guarantee to estimate the better maximum entropy model when adding more constraints.

**Figure 2:**
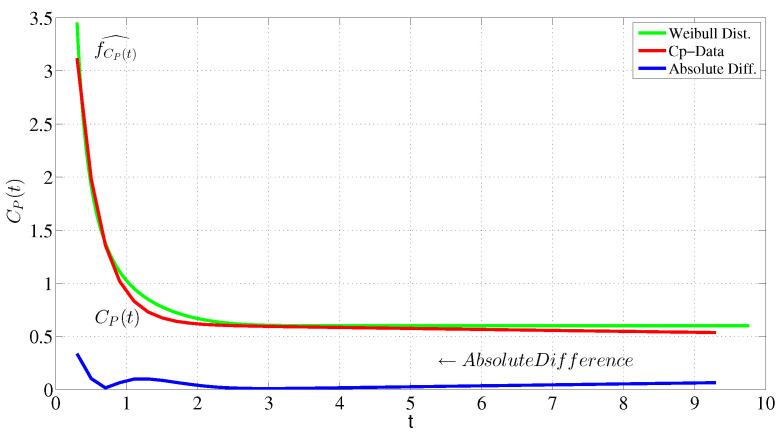
Maximum entropy probability density function of AIF (${\hat{f}}_{C_{p}}\left(t\right)$) and empirical AIF.

After examining several known functions [3] and their exceptions (constraints) which are computed numerically based on Taylor’s theorem from the sample data, the estimated probability density function of the data fits well to the Weibull distribution (Figure 2). For data ${\vec{C}}_{p}\left(t\right)$

$$\begin{array}{ccc} & & \int_{t}f_{C_{p}}\left(t\right)dt=1,\\ & & \int_{t}\log\left(C_{p}\right)f_{C_{p}}\left(t\right)dt\;=-0.4465,\\ & & \int_{t}C_{p}^{3}f_{C_{p}}\left(t\right)dt\;=1.0930 \end{array}$$

using the general form of the maximum entropy distribution Equation (15), ${\hat{f}}_{C_{p}}\left(t\right)$ can be as follows:

$$f_{C_{p}}\left(t\right)=e^{-\lambda_{0}-\lambda_{1}\log\left(C_{p}\left(t\right)\right)-\lambda_{2}C_{p}^{3}\left(t\right)},$$

where the final ME multipliers $\lambda^{\prime }$s and the Weibull parameters are estimated as follows

(27) $${\hat{f}}_{C_{p}}\left(t\right)=\exp\left(-0.7466-1.4944\log\left(C_{p}\left(t\right)\right)-0.1128C_{p}^{3}\left(t\right)\right)+0.5.$$

and based on the ME form of Equation (23)

$$f\left(x\right)=e^{\log\left(\frac{k}{c^{k}}\right)+\left(k-1\right)\log\left(x\right)-{\left(\frac{x}{c}\right)}^{k}}.$$

in which

(28) $$\begin{array}{ccc} \lambda_{0} & = & -\log(\frac{k}{c^{k}}), \end{array}$$

(29) $$\begin{array}{ccc} \lambda_{1} & = & -(k-1), \end{array}$$

(30) $$\begin{array}{ccc} \lambda_{2} & = & c^{-k}. \end{array}$$

Then, according to Equations (27) and (28), the Weibull parameters will be c=1.8498,k=3 where the mean of absolute error, $D_{K-L}$ divergence and entropy are 0.0470, 0.0438, and 0.2026, respectively, see Figure 2.

**Figure 3:**
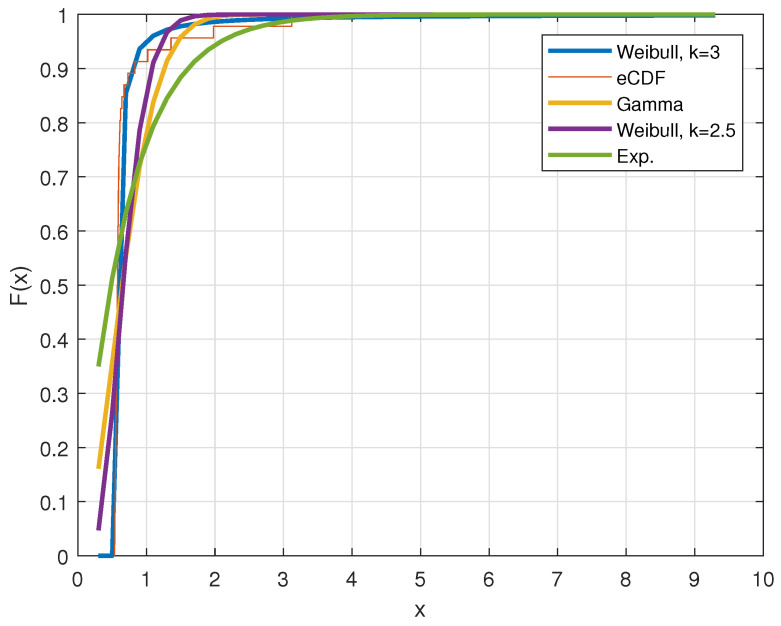
Maximum entropy distribution of AIF, empirical CDF of data and gamma and exponential CDFs.

Table 1 lists the mean absolute error (MAE), Kullback–Leibler distance $D_{K-L}$ and the entropy of different AIF models via MMEM described in Section 2.3 and the empirical one. In each case, we have applied evaluation methods to check the validity of the estimated model. The high measurement of entropy shows the superiority of the Weibull probability density function to fit the data, too. The MMEM can not optimize the different values of *k* and *c* by itself (based on the uniqueness of the maximum entropy distribution [4]), but it can be applied on a grid on *k*. However, using MAE, $D_{K-L}$ and entropy, we achieve different optimal models with the optimal values of their parameters. Additionally, Figure 3 pictures the fit for the different CDFs.

**Figure 4:**
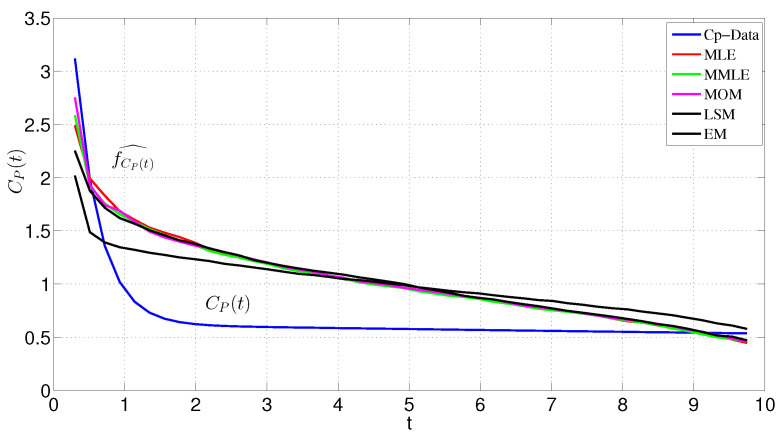
Modified maximum entropy PDF of AIF, empirical AIF, maximum entropy PDFs combined with parameter estimation methods.

Table 2 lists the estimated Weibull parameters via different estimation methods as mentioned in Section 3. All the estimated models are presented in Figure 4. Table 3 indicates the evaluation measurements for all the mentioned method in Section 3 to investigate how the proposed method works. Among all these models, the MMEM has the best fit to the data.

Based on the results of Table 1, Table 2 and Table 3 MMEM has achieved a much better fit model to the data. Actually, Table 2 shows the parameter estimations of the Weibull distribution via different methods in comparison to those via MMEM to see in which case the estimated model fits well to the data. In (Table 3), we examined the results via the root mean square error (RMSE), the goodness of fit ($\chi^{2}$), determination coefficient ($R^{2}$) and the adjust determination coefficient ($R^{2}$) which highlights the MMEM. The proposed MMEM gives the estimation with the lowest absolute error and $D_{K-L}$ divergence with the highest entropy.

## 5. Evaluation

**Figure 5:**
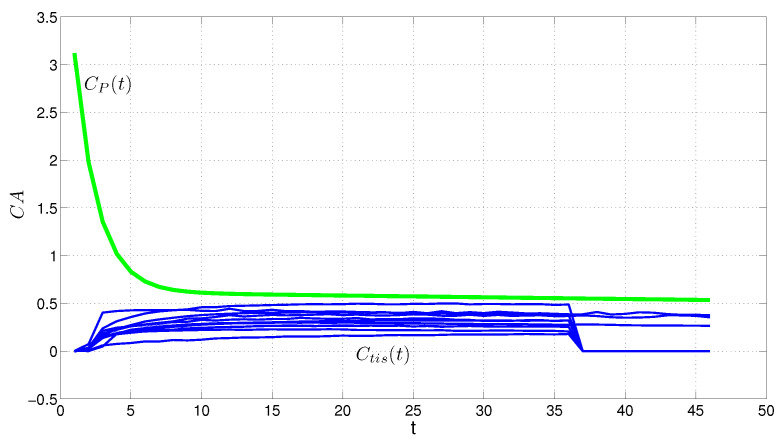
Empirical density model of the contrast agent in plasma ($C_{p}\left(t\right)$) as AIF and tissue ($C_{tis}\left(t\right)$) for 12 patients.

**Figure 6:**
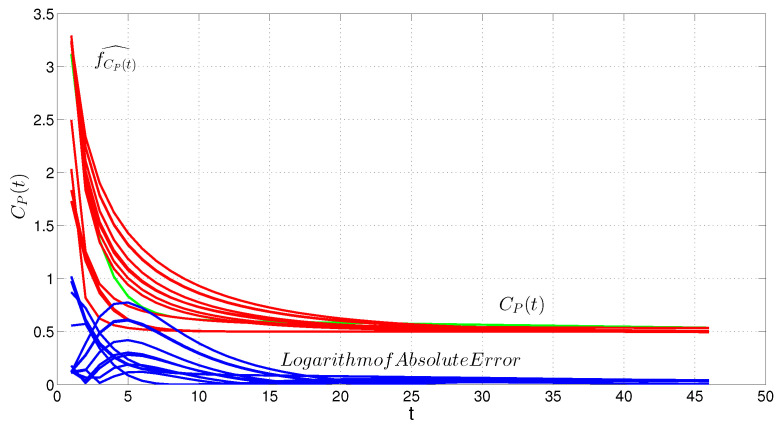
Estimating AIF via MMEM for 12 patients and empirical AIF.

To better evaluate the desired method (MMEM), we considered the data of 12 more patients in total. MMEM and MAP were utilized to estimate the AIF and the kinetic parameters, and the results were compared accordingly, (Figure 5 and Figure 6). The difference within empirical AIF and estimated AIF via the MMEM is clear.

Actually, the AIF in the first two minutes is typically estimated higher than the assumed AIF, whereas there would be negligible difference after about two and a half minutes. However, the correct estimation of the AIF at the onset is the most important for the correct estimation of the kinetic parameters. The K-L divergence measurements range from 0.001 to 0.0637 for all patients.

**Figure 7:**
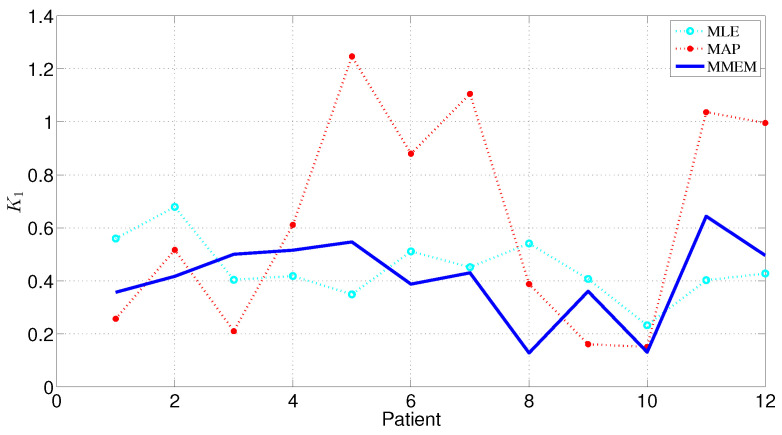
k1 estimated using MMEM/MAP and assumed AIF/ML & MEM/MAP for all 12 patients.

Figure 7 depicts the estimated $k_{1}$ values using MMEM/MAP and assumed AIF/ML & MEM/MAP for all 12 patients. For the MMEM, the *k* values (Table 4) are more on the same level between patients—compared to the MEM/MAP—which biologically makes sense. Still, the AIF is estimated from the data, which makes the estimation of *k* more realistic than the estimation using an assumed AIF.

## 6. Discussion and Conclusions

The main purpose of this study is to connect an important problem in statistics which is to determine the probability density function of a random variable based on observations to an important problem in image processing which is to determine the AIF in situations where not enough information about the AIF is available and then accurate estimation of the kinetic parameters. In recent years, various parametric and non-parametric methods have been introduced for estimation of the probability density function for a random variable based on observations, but there is very limited work reported on the optimization methods.

Therefore, we have introduced a new algorithm which is the combination of MEM/TLBO named MMEM. The maximum entropy method (MEM) is one of the major and strong methods for estimating and determining the probability density with a high level of accuracy and efficiency and minimum bias. The core idea of this approach is to determine the statistical models agree with data. In other words, the MEM provides a method to find the least biased model that is consistent with the data, i.e., the maximally noncommittal with regard to missing information [1,2,3,4].

A number of calculations and comparisons have been conducted for the estimation of the AIF and the kinetic parameters, respectively, (Figure 5, Figure 6 and Figure 7 and Table 1, Table 2, Table 3 and Table 4 ). The results have revealed the characteristics of the empirical PDF of AIF as well as the fact that the modified maximum entropy approach performs adequately in fitting an appropriate model by comparing with the Weibull distributions to the AIF with consideration of its accuracy and applicability.

The aim of this work is not exactly to make decisions on the AIF or Cp, but it is very important how exactly the accurate kinetic parameters are determined. These parameters are so significant and are of clinical relevance, for that, clinicians can easily interpret them. Using the MMEM/MAP guarantees to minimize bias in the estimation of the AIF and the kinetic parameters

Since the AIF plays an important role in the analysis of DCE-MRI, in cases where the AIF could not be determined in the image, the literature AIF is a standard technique. The proposed method in this study gives an alternative way to assess the input function from the existing data. We have shown that the proposed method allows a good fit of the data and a good estimation of the kinetic parameters.

To further evaluate this method, we propose to apply it on terms of energy efficiency and system complexity. 

## Figures and Tables

**Table 1:** Comparison of the estimated maximum entropy AIFs, and the empirical AIF.

Estimated Distribution	MAE	$D_{K-L}$	Entropy
Gamma	0.0775	0.0285	0.0303
Exponential	0.0375	0.0363	0.0872
Weibull (k=3)	0.0470	0.0438	0.2026
Weibull (k=2.6)	0.0403	0.0389	0.1755
Weibull (k=2)	0.0471	0.0342	0.1471

**Table 2:** Weibull parameters via different parameter estimation methods.

*Methods*	*K*	*C*
EM	1.6469	0.7787
MOM	1.9125	0.7850
MLE	1.8005	0.7890
MMLE	2.0201	0.7758
NLSM	2.7767	0.7518
MMEM	2.6	1.7380

**Table 3:** Evaluating methods to compare the empirical AIF and the maximum entropy PDFs of AIF.

*Methods*	*RMSE*	*Chi-Square*	$R^{2}$	Adjust $R^{2}$
EM	0.286	0.0755	0.631	0.622
MOM	0.255	0.0691	0.670	0.663
MLE	0.278	0.1191	0.570	0.580
MMLE	0.274	0.0771	0.636	0.628
NLSM	0.194	0.2854	0.535	0.525
MMEM	0.0320	7.5687 × 10${}^{-4}$	0.995	0.995

**Table 4:** Kinetic parameters estimation via MMEM/MAP for 12 patients.

Patient	1	2	3	4	5	6
$k_{1}$	0.1637	0.1016	0.7175	0.1650	0.5959	1.0477
$k_{2}$	0.0210	0.3688	0.1073	0.2079	0.1233	0.0072
**Patient**	**7**	**8**	**9**	**10**	**11**	**12**
$k_{1}$	0.6309	0.7980	0.1085	0.4327	0.544	1.0225
$k_{2}$	0.0701	0.3861	0.2377	0.0839	0.235	0.0271

## Data Availability

Patient data are not made available.
